# Landscape complexity effects on *Brassicogethes aeneus* abundance and larval parasitism rate: a two-year field study

**DOI:** 10.1038/s41598-023-49690-1

**Published:** 2023-12-16

**Authors:** Silva Vilumets, Riina Kaasik, Marjolein Lof, Gabriella Kovács, John Holland, Eve Veromann

**Affiliations:** 1 Plant Health Chair, Estonian University of Life Science, Kreutzwaldi 1, 51006 Tartu, Estonia; 2 Environmental Systems Analysis Group, Wageningen University and Research, 6708PB Wageningen, the Netherlands; 3 Game and Wildlife Conservation Trust, Fordingbridge, Hampshire, SP6 1EF UK

**Keywords:** Ecosystem services, Agroecology

## Abstract

Global biodiversity has suffered a decline primarily attributed to landscape simplification and intensified agricultural practices. Agricultural environments, characterized by homogeneity and frequent disturbances, are often suboptimal habitats for various insect species. While agricultural fields do favour pests, they generally fail to provide suitable habitats for natural enemies. The inclusion of diverse supporting habitats, such as semi-natural habitats, grassy and woody field margins etc. surrounding agricultural fields, play a crucial role in fostering effective biodiversity conservation. Moreover, determining the influence of different adjacent habitat types is essential in elucidating their influence on pest abundance and parasitism rates. Our two-year field study focused on assessing the abundance of *Brassicogethes aeneus* and its parasitism rate. The findings revealed that the adjacent habitat type did not significantly increase pest abundance and the parasitism rate of *B*. *aeneus* larvae consistently stayed over the threshold for effective biological control throughout the fields. This was attributed to the high proportion (35 and 38% in the 2 study years) of semi-natural habitats within most of the 1 km radius study areas. While our study did not identify any specific adjacent habitat type or habitat within a 1 km radius that directly impacted *B*. *aeneus* abundance, it emphasises the intricate interplay between the pests, parasitism and the surrounding environment because the interactive effect of distance from the crop edge and habitat type had a significant influence on *B*. *aeneus* infestation levels but not on parasitism. Decision tree analysis suggests that > 18% semi-natural habitat is needed to ensure sufficient levels of parasitism for effective biological control. A comprehensive understanding of habitats that influence not only *B*. *aeneus* but also other pests is critical for the successful implementation of IPM strategies and conservation initiatives within the agricultural sector.

## Introduction

The global biodiversity crisis has worsened in the last decades, mostly due to landscape simplification and the intensification of the agricultural sector^1–3^. To compensate for the biodiversity decline surrounding agricultural fields, targeted agri-environment schemes have been implemented, supporting farmers in adopting organic farming practices, by adding more flowering strips and grassy field margins to the agricultural landscape^4,5^. While implementing agri-environmental schemes on farmland offers advantages, it is crucial to retain semi-natural habitats (SNH) around the agricultural fields for effective biodiversity conservation. SNHs provide necessities, like food resources, shelter, and hibernation habitats, for various insect taxa^6^, including pollinators and predatory arthropods. Pollinators and predatory arthropods play vital roles in agricultural production by increasing yield through pollination and reducing pest density through biocontrol^7–11^.

Oilseed rape (*Brassica napus* L.) (OSR) is an economically important crop around the world, mainly used to produce vegetable oil and biofuel feedstock, grown in large monocultural fields, and attracts various herbivorous insects specialised on cruciferous plants. One such insect that causes damage to OSR plants is the pollen beetle (*Brassicogethes aeneus* Fabricius syn. *Meligethes aeneus* Fabricius), damaging both winter (WOSR) and spring oilseed rape plants at the green bud stage that may cause yield reduction. *Brassicogethes aeneus* overwinter in woody and sheltered habitats and in spring, they seek food sources and oviposition sites^12^. They oviposit into the buds, and while first instar larvae develop inside the buds, the second instar larvae exclusively feed on pollen from open flowers^13^. However, the main damage is done by adults feeding on the buds, leading to bud abortion and eventual cessation of seed development^14^, resulting up to 80% loss in yield on spring oilseed rape^15^. The control of *B*. *aeneus* in conventional OSR fields relies on synthetic insecticides, which not only reduces the abundance of the target pest but also affects the natural pest control providers and pollinators^16–19^. In line with the European Union’s Farm to Fork Strategy, there is a target to reduce overall pesticide use by 50% by 2030^20^. This urges farmers to adopt alternative pest control methods and rely on the predatory arthropods and parasitoid wasps. Parasitism by parasitoids play a significant role in natural pest control. *Brassicogethes aeneus* is attacked by at least nine species of hymenopteran parasitoids, with three key species: *Tersilochus heterocerus* (Thomson), *Phradis interstitialis* (Thomson) and *Phradis morionellus* (Holmgren)^21^. These three parasitoid species are univoltine and koinobiont endoparasitoids. They overwinter in pupal cocoons in the soil, close to their hosts, and emerge in spring to migrate to the OSR fields^22^. It is known that these parasitoids differ in their host-finding behaviours and attack their hosts at different growth stages, suggesting niche separation among them^23^. Despite the host-finding behaviours, studies have shown that the parasitism rates of *B*. *aeneus* can vary widely from 0 to 63%^24–26^, and can depend on the distance from adjacent habitat^27^ and farmland management methods^28–30^. Landscape structure and composition significantly affect both *B. aeneus* and its parasitoids. One alternative pest control method is conservation biological control. This aims to reintroduce beneficial insects into crop systems to improve natural pest management. Previous studies have highlighted the links between conserving natural habitats and reducing pest pressure in farmland^31,32^. Whereas homogeneity in the landscape enhances the presence of *B*. *aeneus* in OSR field, more heterogenic landscape enhances species richness, abundance and fecundity of parasitoid wasps^7,32,33^. Additionally, complex landscapes, as opposed to simple ones, provide a higher parasitism rate of *B. aeneus*^7,34^. *Brassicogethes aeneus* abundance is influenced by changes in total OSR area size and the distance from the previous year’s OSR fields, which can effectively lower *B*. *aeneus* abundance while maintaining sufficient parasitism rates^26^. In this two-year study, we examined the landscape effects on *B. aeneus* abundance and parasitism of *B. aeneus* larvae in OSR fields. Our objectives were to determine the influence of adjacent habitat type and the in-field distance from the adjacent type on the abundance and parasitism rate of *B. aeneus*. Additionally, we sought to examine how the surrounding habitat types within a 1 km radius affect both *B. aeneus* abundance and the parasitism rate of *B. aeneus* larvae.

## Results

### Landscape characteristics

In 2014 and 2015, across the studied landscape circles the average coverage of arable land was 53.8% and 58%, respectively, with a minimum coverage of 20.2% and a maximum of 82.7%. The average SNH proportion across landscape circle coverage was – 37.7% in 2014 and 34.7% in 2015, the minimum cover of SNH was 11.3% (in 2015) and maximum of 52% (in 2015), of which woody areal elements typically occupied the largest area. The average coverage of OSR fields was 14.7% in 2014 and 15.7% in 2015. The mean proportion of SNH in landscape circles of focal fields with different adjacent elements was quite similar in both study years—herbaceous linear element [containing narrow (1.5–12 m width) grassy crop margin] 33.8%, woody linear element (containing hedge or line of trees) 40.9%, and another crop field (either bordering with OSR, barley, wheat, potato, oat, or pea) 38.3% in 2014 and 33.8%, 36.4% and 34.2% respectively in 2015. Also, the mean proportion of arable land in landscape circles was relatively similar in both years. In 2014, arable land constituted 54.0% on landscape circles where focal field was bordered by herbaceous linear element, 51,6% on landscape circles where focal field was bordered by woody linear elements and 55.9% on landscape circles where focal field was bordered by another crop field In 2015, the proportions changed slightly – 59.3%, 54.6% and 60.1%, respectively. The full list of landscape characteristics is presented in the supplementary materials (Table S1–S2).

### Effects of adjacent habitats and distance from the edge of the field on B. aeneus abundance

**Figure 1:**
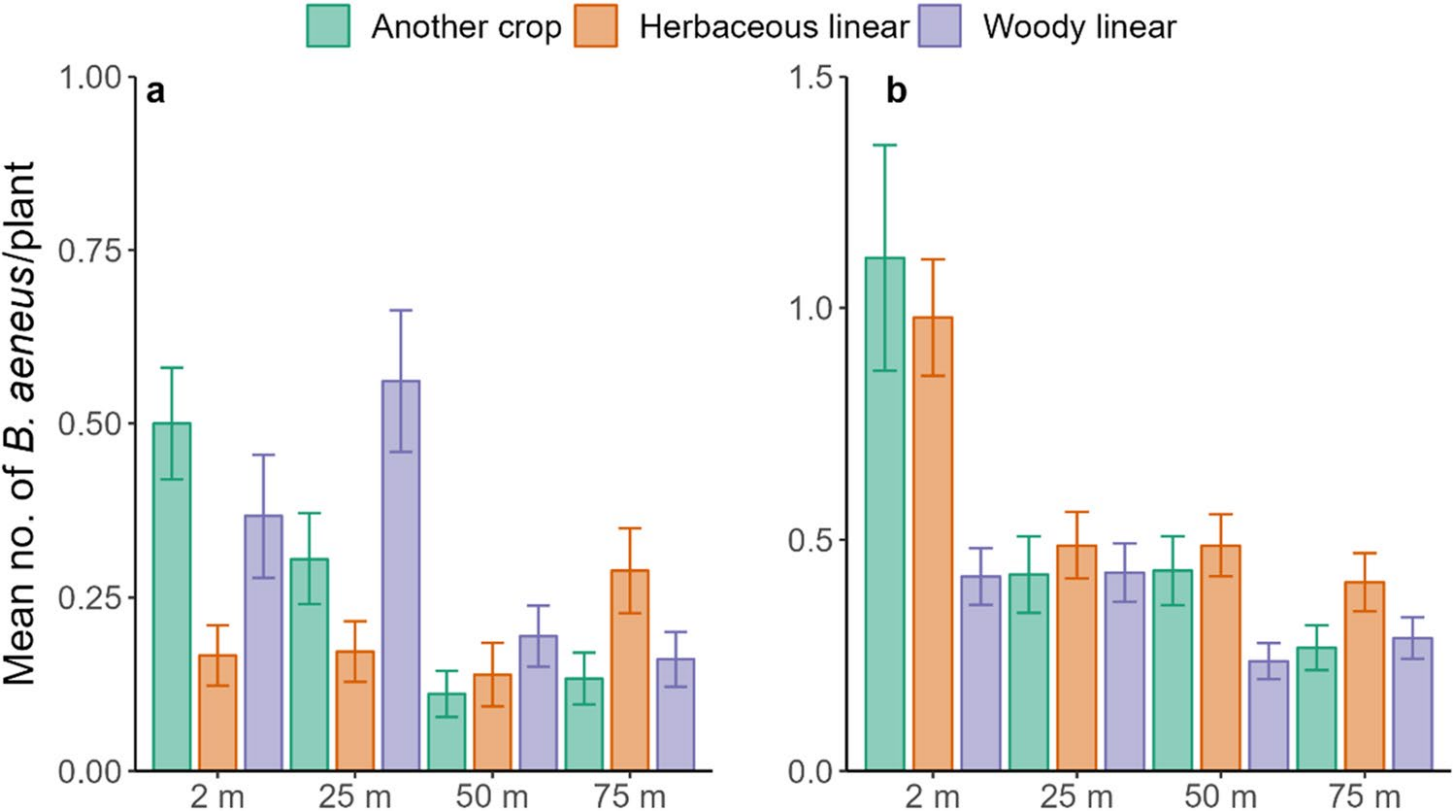
Mean (± SE) number of *Brassicogethes aeneus* adults per plant during the bud stage of WOSR (BBCH 50–59) collected from WOSR fields with different adjacent habitat types and from different distances from the edge of fields in 2014 (**a**; N = 216) and 2015 (**b**; N = 288). The probabilities from the variance analysis: 2014: *P*_distance_ < 0.0001, *P*_adjacent habitat_ = 0.28, *P*_distance*adjacent habitat_ < 0.0001; and 2015: *P*_distance_ < 0.0001*P*_adjacent habitat_ = 0.09, *P*_distance*adjacent habitat_ < 0.0001.

Generally, throughout the study period, the mean number of *B*. *aeneus* per WOSR plant was low and stayed firmly under the economic threshold level (in Estonia 1–2 beetles per plant at BBCH 50–51 and 3–5 at BBCH 55–59^35^). The mean number of *B*. *aeneus* adults was high and surpassed the economic threshold level (mean 5.72 ± SE 1.29) in only one of the fields, during the last sampling round (BBCH 55–59) in 2015. There were three sampling rounds in 2014, during which a total of 2160 WOSR plants were tapped, and four sampling rounds in 2015, during which a total of 2880 WOSR plants were tapped. Observing the yearly means of *B*. *aeneus* per study field, shows that only one field in 2015 was close to the economic threshold, when the mean number of beetles per plant was 1.86 (SE ± 0.37) (Table S3). In 2014, the mean number of *B. aeneus* per plant was highest in fields adjacent to woody linear (0.32 ± SE 0.79), followed by another crop (0.26 ± SE 0.56) and herbaceous linear habitat (0.19 ± SE 0.39) (Figure 1). Whereas in 2015, the greatest mean number per plant was found in fields adjacent to herbaceous linear (0.59 ± SE 0.84), followed by another crop (0.56 ± SE1.54) and woody linear (0.34 ± SE 0.41) (Figure 1). The combined effect of distance and adjacent habitat type (2014: χ^2^= 65.35, df= 6, *p *< 0.0001; 2015: χ^2^= 43.76, df= 6, *p *< 0.0001) had a significant effect on the estimated mean abundance of *B. aeneus* adults in both study years. Generally, the estimated mean value of *B. aeneus* per ten plants was higher at 2 m from the adjacent habitat and the abundance declined with distance from the boundaries (Table 1).

**Table 1:** Differences in the estimated mean number of *Brassicogethes aeneus* adults per plant during the bud stage of WOSR (BBCH 50–59) collected from WOSR fields with different adjacent habitat types and from different distances from the edge of fields in 2014 and 2015.

Year	Another crop				Herbaceous linear			Woody linear		
	Contrast	df	t	*P*	df	t	*P*	df	t	*P*
**2014**	2 m versus 25 m	201	2.877	**0.0219**	201	− 0.128	0.9992	201	− 2.688	**0.0376**
	2 m versus 50 m	201	6.084	** < .0001**	201	0.673	0.9064	201	3.034	**0.0138**
	2 m versus 75 m	201	5.753	** < .0001**	201	− 2.407	0.0784	201	3.691	**0.0015**
	25 m versus 50 m	201	3.874	**0.0007**	201	0.800	0.8532	201	5.403	**< .0001**
	25 m versus 75 m	201	3.390	**0.0044**	201	− 2.288	0.1034	201	5.923	**< .0001**
	50 m versus 75 m	201	− 0.602	0.9288	201	− 3.017	**0.0153**	201	0.749	0.8742
**2015**	2 m versus 25 m	273	8.230	** < .0001**	273	6.164	** < .0001**	273	− 0.140	0.9990
	2 m versus 50 m	273	8.120	** < .0001**	273	6.164	** < .0001**	273	3.453	**0.0034**
	2 m versus 75 m	273	10.232	** < .0001**	273	7.273	** < .0001**	273	2.440	0.0714
	25 m versus 50 m	273	− 0.139	0.9990	273	0.000	1.0000	273	3.584	**0.0025**
	25 m versus 75 m	273	2.923	**0.0189**	273	1.294	0.5644	273	2.575	0.0506
	50 m versus 75 m	273	3.056	**0.0129**	273	1.294	0.5644	273	− 1.067	0.7082

*df* Degrees of freedom, *t* t statistic, *P P* value.

*P* values in bold show statistically significant differences (*p* < 0.05).

### Effects of habitats surrounding the focal field on B. aeneus abundance

We analysed the correlations between *B. aeneus* abundance in focal fields and various landscape characteristics within 1 km radius from the focal field in pooled data of 2014–2015. In our study, we did not find any significant correlations between them. Furthermore, we also carried out random forest tree analyses but none of the forests could describe representative proportion of the samples. The best model described only 16% from the samples that is not enough to extrapolate these results to the whole population and to draw fundamental conclusions. However, only for 2014 a positive correlation between herbaceous linear elements and the abundance of *B. aeneus* was found (Pearson correlation r = 0.68; *p* = 0.002; Table S4). In 2014 the abundance of *B. aeneus* increased significantly with increasing proportion of herbaceous linear habitats within the 1 km radius of focal field however, in the next year, 2015, the correlation between herbaceous linear habitats and *B. aeneus* abundance was opposite, meaning the abundance of *B*. *aeneus* decreased with increasing proportions of herbaceous linear habitats but relationship was not statistically significant. Therefore, we cannot conclude that herbaceous linear habitats may increase *B. aeneus* abundance as our results were inconsistent. Other habitats within the 1 km radius of focal field did not have significant correlations with *B. aeneus* abundance, in both 2014 and 2015 (Tables S4–S5).

### Effects of adjacent habitats and distance from the edge of the field on parasitism rate

**Figure 2:**
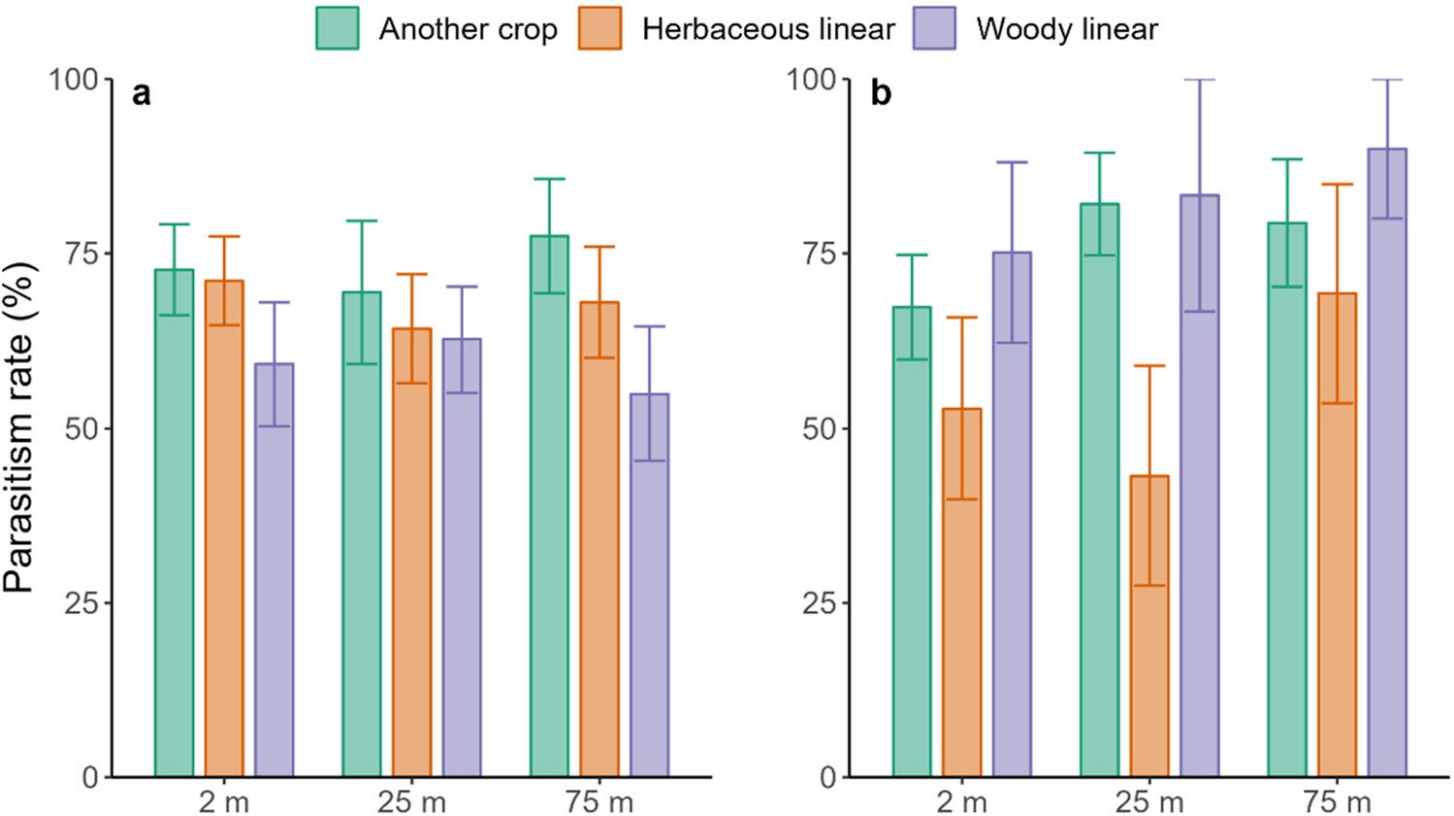
Mean (± SE) parasitism rate of *Brassicogethes aeneus* on WOSR field, depending on the distance from the field edge and the adjacent habitat type in 2014 (**a**; N = 162) and in 2015 (**b**; N = 162). The probabilities from the variance analysis: 2014: *P*_distance_ = 0.917 *P*_adjacent habitat_ = 0.467, *P*_distance*adjacent habitat_ = 0.128; and 2015: *P*_distance_ = 0.407 *P*_adjacent habitat_ = 0.583, *P*_distance*adjacent habitat_ = 0.108.

The parasitism rate of *B*. *aeneus* larvae in both study years was very high and even reached up to 100% at field level on three fields adjacent to herbaceous linear and woody linear habitats, while in two focal fields adjacent to herbaceous linear habitat no parasitism was observed (Table S3). The parasitism rate in both study years was unaffected by adjacent habitat, distance from the adjacent habitat nor the combined effect of them (all *p* values > 0.05) (Figure 2). The mean parasitism rate in 2014 and 2015 was high with 66.24% (SE ± 2.71) and 71.85% (SE ± 3.87), respectively. The mean parasitism rate was high across all fields regardless of the adjacent habitat, both in 2014 and 2015. The highest parasitism rate was observed in focal fields adjacent to another crop 73.04% (SE ± 4.73) in 2014 and in 2015 in focal field adjacent to woody linear habitat 81.02% (SE ± 7.89).

### Effects of landscape characteristics on B. aeneus parasitism rate

**Figure 3:**
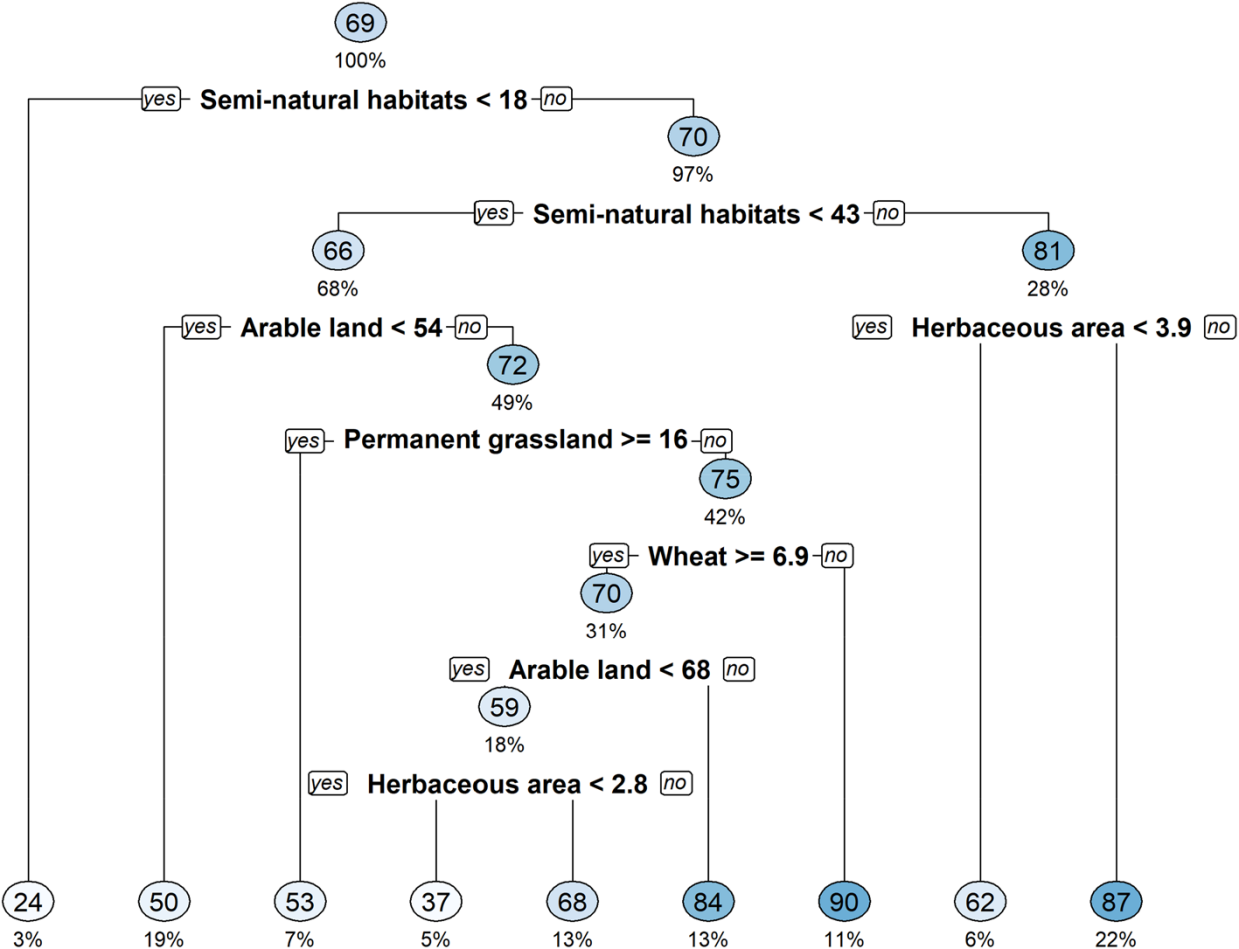
Decision tree based on the 2014 and 2015 landscape characteristics surrounding the focal fields to determine the median parasitism rate of *Brassicogethes aeneus* (pooled data 2014 and 2015). Each terminal node (inside the circle) gives a predicted median parasitism rate of *B*. *aeneus* and under the node the value [percentage (%)] represents the proportion of data used to predict the parasitism rate. The values behind the landscape variable represent the percentage, which was used for the division setpoint.

The two years parasitism rate of *B*. *aeneus* was very high and reached up to 69%. The random forest analysis showed an overall R-squared value of 0.45 suggests that approximately 45.3% of the variance in the *B*. *aeneus* parasitism percentage can be explained by the predictor variables selected by the model. A decision tree (Figure 3) revealed that the parasitism rate of *B*. *aeneus* varied based on the landscape characteristics surrounding the focal field. When the semi-natural habitat proportion (SNH) around the focal field was below 18%, the parasitism rate was only 24%. When the SNH proportion around the focal field was over 18%, the predicted parasitism rate was 70%. It is important to highlight that 97% of data connected with parasitism rate were sampled from landscape circles where SNH proportion was greater than 18% and only 3% of data was collected from landscape circles with SNH percentage below 18%. In addition, almost 30% of parasitism rate data was collected from areas where SNH proportion was greater than 43%. Thus, our data reflect an agricultural landscape, that is heterogeneous due to its very high SNH coverage, which is important for conservation biological control. The decision tree also revealed that if the proportion of SNHs exceeded 43% and the proportion of herbaceous area was over 3.9% in the surrounding landscape then the median of parasitism rate of *B*. *aeneus* was 87%. When proportion of SNHs was lower than 43% then the proportion of arable land influenced the parasitism rate—resulting in higher parasitism rate (72%), if the proportion of arable land was more than 54%, whereas if the proportion of arable land was smaller than 54%, the parasitism rate was 50%.

## Discussion

The field study conducted over two consecutive years showed very low abundance of *B. aeneus* during the damage susceptible growth stage of WOSR and even though no relation between the bordering habitat and their abundance was found. The mean larval parasitism rate of *B*. *aeneus* was extremely high with 69% and occasionally reaching up to 100%, indicating an effective natural pest control in the landscape. This is confirmed by the decision tree that showed that landscapes with more than 18% SNH coverage have a high (70%) predicted parasitism rate.

The study showed that the abundance of *B. aeneus* during WOSR bud stage, that is the growth stage most susceptible to damage by *B. aeneus*, was very low in both years and the bordering habitat had no significant impact on their abundance. Also, it is important to highlight that in our study area the agricultural landscape surrounding study fields had very high proportion of SNHs within 1 km radius around the focal fields (Table S3). Therefore, our study shows that in diverse agricultural landscapes comprised of woody and herbaceous linear elements there is a lower risk of *B*. *aeneus* infestations in WOSR. This was despite the presence of a multitude of overwintering sites near the focal fields. Thus, the number of *B*. *aeneus* was low and did not exceed the economic threshold during bud stage of plants. Studies on the *B*. *aeneus* density, plant damage, and local or regional landscape effects were shown to be inconsistent. Several studies suggest that complex landscapes may reduce pest density^34,36–41^, although there are also evidence of increasing pest pressure^42,43^. The proportion of woodland in the landscape is usually associated with an increase of *B*. *aeneus* abundance^43–45^, whereas for grasslands there were contrasting results, indicating both increases and decreases in abundance^34,42^. As woody areas provide a suitable overwintering habitat for *B*. *aeneus*, this can increase pest density in the spring^46^, however, regardless of presence of quite large woodland areas in the study sites, the results of our study did not confirm this finding as we did not find any significant correlations between beetles abundance and woodland proportions. Pest density and its relationships with the landscape configurations have complicated and multilevel ties. There are many direct and indirect factors at large and small scale that influence pest abundance such as crop rotations, pesticide usage, tillage regimes, abundance, and species richness of naturally occurring enemies of pests, pest pathogens, amount, quality and connectivity of landscape elements, soil health, weather conditions etc. In our study, the adjacent habitat of WOSR did not influence *B*. *aeneus* abundance, but the distance from the adjacent habitat and the combined effect of distance and adjacent habitat type had a significant impact on the estimated mean abundance of *B*. *aeneus* throughout the study years. The abundance of *B. aeneus* during inflorescence emergence stage (BBCH 50–59) was greater at 2 m and 25 m from the edge of the WOSR and decreased at 50 m and 75 m from edge. This suggests that local adjacent habitats were the source of infestation. It was not possible to identify which habitat type led to greater infestations because results were inconsistent between years. Other studies also showed that *B*. *aeneus* tends to aggregate on the field edge during bud stage of plants and expand more into the field once the plants start to flower^47–49^. This shows that there are opportunities to use Integrated Pest Management (IPM) tools such as a precision agriculture techniques and/or trap cropping to manage *B. aeneus* abundance and use insecticide treatments only along WOSR headlands to reduce the agrochemical inputs and preserve natural enemies^24,25,48,50–52^. However, aggregation at the edges of fields is not common for all insect pests of oilseed rape, for instance cabbage seedpod weevil (*Ceutorhynchys obstrictus* Marsham), does not have similar in-field immigration pattern and is evenly distributed at different distances from the edge^53^, also *Psylliodes chrysocephala* (L.) is non-uniformly distributed within the crop and does not aggregate along field edges^54^. This may because of differences in overwintering sites and/or dispersal abilities.

In both years, the parasitism rate of *B. aeneus* larvae was extremely high with average rate of 69%, which is close to previous finding in Estonia^26^ and over the minimum effective biological control level (at least 32%)^55^. In our study, we found that the parasitism rate was not influenced by the adjacent habitat or by distance from the field edge. Interestingly, the mean parasitism rate was consistently high across almost all focal fields regardless of the adjacent habitat type, indicating a strong and efficient level of natural pest control against *B*. *aeneus* in both years. Our findings are comparable to Kovács et al.,^53^, where they also showed a similar effect on *C. obstrictus*; the adjacent habitat type did not affect the parasitism rate which was likewise outstandingly high. In our study, in 33 of 36 fields the parasitism rate was above the threshold value of effective biological control (32–36%)^55^, even reaching up to 100% in three fields. Thus, the population size of beetles was efficiently controlled by hymenopteran parasitoids. Unfortunately, it was not possible to identify the impact of adjacent habitats on levels of parasitism in focal fields. The high proportion of different semi-natural and other non-cropped habitats can potentially provide suitable habitats for parasitoids and created an abundance of parasitoids at a landscape scale, thereby masking any field-scale effects. Landscape complexity and the amount of SNHs in agricultural landscape have previously shown to have a positive impact on pollen beetle (*Brassicogethes* ssp) parasitism^34,37,41,56,57^. Semi-natural habitats around and nearby arable fields provide alternative host plants^32,58–60^, and as SNHs tend to have lower disturbance, parasitoids can sustain a proficient population at landscape level and therefore provide the pest control service to agricultural fields. Furthermore, the study by Le Clec'h et al.^61^ indicated a dilution of the additional effect of linear elements on ecosystem services supply when linear elements were implemented in a landscape where the supply of ecosystem services was already high, compared to a landscape where the initial supply was low. The decision tree created in our study, based on the landscape and parasitism data, revealed that if the SNH percentage was below 18% in the 1 km radius landscape circle the parasitism rate was low. Thies and Tscharntke^60^ showed that the SNH coverage below 20% led to the decrease of *B*. *aeneus* parasitism below the threshold necessary for effective biological control. The decision tree based on our data confirms their results—our model predicted that if the percentage of SNHs was below 18% then the predicted parasitism rate was only 24% and if it was over 18% then predicted parasitism rate was 37–90%. Thus, our results show that having a complex landscape, it is possible to provide a consistent parasitism rate over the two years. Furthermore, two main parasitoid species of *B*. *aeneus*, *Phradis morionellus* and *P. interstitialis* are oppositely affected by the woodland coverage around OSR fields^62^ and may therefore contribute differently on the overall parasitism of *B. aeneus* based on woody habitat abundance and location. To ensure functioning ecosystem, pollination, biological pest control service at least 10–20% SNH is recommended per km^2^, although our results suggest that 20% would be the minimum level in 1 km radius of a focal field^63^. Our results also support the previous findings showing that the landscape with a low proportion of oilseed rape had a negative effect on the parasitism rate^37^. In the same study region, Kovács et al.,^53^, stated that the landscape in the study area is still sufficiently diverse and complex, providing different habitats for both the pest and the natural enemies. Our results classified by the decision tree highlight the importance of landscape characteristics, particularly the presence of SNH as these types of areas are the necessary for conservation biocontrol.

In conclusion, our study highlights the complicated relationship between pest, parasitoids and landscape and highlights that a specific adjacent habitat type or the habitat within the 1 km radius did not increase *B*. *aeneus* abundance. Understanding the habitats and factors that influence not only *B*. *aeneus* but also other pests, is vital for implementing successful integrated pest management strategies and conservation efforts in agricultural landscapes. Future study should investigate the processes that drive parasitism rates and evaluate other factors that may contribute to the observed trends. Such knowledge will be useful in designing efficient strategies to improve natural enemy populations and facilitate biological control of *B. aeneus* in agricultural landscapes.

## Methods

The study was carried out in 18 non-overlapping landscape circles with a radius of 1 km around the focal fields in Tartu County, Estonia in 2014 and 2015. General study site selection, landscape circle radius of 1 km, classification of SNH-s and experimental design were based on the standardized protocols of the EU FP7 project QuESSA across participating regions and crops and are previously described in detail:^53,64–67^. For the project as well as our study, the 1 km radius was chosen, as it aligns with the recommended spatial scale for the dispersal of oilseed rape parasitoids in landscape sectors^41,62^. In both study years, 18 WOSR focal fields were selected; six bordered with woody linear, six fields with herbaceous linear and six with another crop. The minimum size of the focal field was 3 ha, the width for both woody linear and herbaceous linear was between 1.5 and 25 m and at least 50 m long. In 2014, the bordering crop was spring oilseed rape (1 field), barley (2 fields), wheat (2 fields), potato (1 field), and in 2015 we had barley (1 field), wheat (2 fields), oat (1 field), pea (2 fields). The landscape surrounding the focal field within 1 km radius for both years (2014 and 2015), was mapped separately with ArcMap 10.1 (ESRI 2012) using data from the Estonian Land Board and the Agricultural Registers and Information Board with any remaining gaps filled during field visits. The minimum distance between fields was larger than 1 km, ensuring that the landscape in the 1 km radius around the focal field did not overlap. Based on the dominating vegetation, semi-natural habitats (SNH) were classified as: herbaceous areal (semi-) natural hayfields or abandoned fields that have not developed > 30% shrub/tree canopy cover), woody areal (woodland), cover crop, herbaceous linear (narrow grassy crop margin) and woody linear (hedge, line of trees), their area and proportion within 1 km radii of the focal field was calculated using ArcMap 10.1. The focal fields in this study were managed conventionally. Each sampling transect was set at 90° from the focal field bordering element progressing into the crop. Within each field they were located at least 25 m away from other field edges, that were not the focal adjacent habitat type.

### B. aeneus abundance assessment

The abundance of overwintered adult *B. aeneus* in WOSR fields was assessed using the plant tapping method^68–70^. The abundance of *B. aeneus* adults was the assessed along the sampling transect at four distances: 2, 25, 50, and 75 m from the edge of the crop. At each sampling point ten random WOSR plants were selected, the plants’ main raceme was tapped over a tray to dislodge *B. aeneus* from the buds and flowers. Dislodged *B. aeneus* which fell onto the tray were counted. In 2014, sampling started at the bud stage of plants (growth stage BBCH 52–53; following the decimal code by Lancashire et al.^71^), and ended when the plant growth stage was BBCH 57–60 (end of bud stage and beginning of flowering stage). In total, three sampling occasions were performed in 2014. In 2015, there were four sampling occasions, sampling started when the plant growth stage BBCH 50–51(bud stage of plants) and sampling ended when the plant growth stage was BBCH 60–61 (start of flowering stage).

### Collection of the B. aeneus larvae and parasitism rate

The parasitism rate of *B. aeneus* larvae was assessed using the funnel trap method^27^. Funnel traps were placed along the sampling transect at three distances: 2 m, 25 m, and 75 m from the edge of the field. The trap consisted of a plastic funnel (diameter 31 cm) and a plastic container (50 ml) attached to the funnel end. The upper part of the funnel trap remained above the ground below the side shoots while the lower part with collection cup was dug into the ground. The trap was placed close to the WOSR plants at the end flowering stage of plants (BBCH 67) and were kept in the field until the end of flowering (BBCH 70). In each year a total of 162 samples were collected. The larvae of the last growth stage were collected as they were ready to leave from flowers and drop to the ground to pupae in soil. The area of which the larvae dropping from the plants were collected was equal to the diameter of the funnel (754.4 cm^2^). Funnel traps were emptied once a week for a period of three weeks. The content of the funnel traps was strained through a fine mesh fabric and placed into labelled plastic bags. In the laboratory, samples were sorted, all *B. aeneus* larvae were counted, placed in labelled Eppendorf tubes, containing distilled water, and stored in the freezer at − 20 °C for later dissection. All larvae were dissected using green food colouring as contrast medium to detect parasitoid eggs and larvae. All parasitoid eggs and larvae were counted and, when possible, identified to species or genus using identification key by Osborne^72^.

### Statistical analyses

Statistical analyses were carried out in R statistical software (version RStudio 2022.02.3 + 492)^73^. To determine the effects of adjacent habitat type and the distance from the field edge on *B. aeneus* abundance (count data), generalized linear mixed model (package “glmmTMB”^74^), with Poisson distribution and log link function was used. We generated a model for both 2014 and 2015, were the adjacent habitat type (categorical variable), distance (categorical variable), and their interaction as fixed-factors and the field site (in total 18 fields per year), sampling round and the number of days from the last insecticide application as random factors in model. Analysis of deviance of Wald statistic Type II was used to test the individual and combined effect of adjacent habitat type and the distance from the edge. We applied estimated marginal means (package “emmeans”^75^) to compare marginal mean of *B*. *aeneus* abundance in the interaction model. Pearson's correlation to examine the relationship between the habitats within the 1 km radius of focal field and *B. aeneus* abundance both in 2014 and 2015 were performed. The parasitism rate of *B*. *aeneus* larvae was determined for each sampling point by multiplying the total number of parasitized larvae by 100 and dividing the result by the total number of larvae captured with the funnel trap at a specific sampling point. To analyse the adjacent habitat type and the distance from the field edge effect on parasitism rate, generalized linear mixed model with binomial distribution was performed, followed by analysis of deviance applying Wald statistic Type II. Adjacent habitat type, the distance from the edge and the combined effect of them were used as fixed effects, and the field number as random factor. Missing values of parasitism rates were removed prior to the analysis. We used DHARMa package^76^ to assess the model fit.

Random forest analysis was carried out to determine the variance that can be explained by the landscape types surrounding the focal fields and afterwards a decision tree was built. For this analysis we combined both 2014 and 2015 dataset of landscape characteristics and the parasitism rate to have complete dataset. For random forest, we used previously described proportions of different landscapes within 1 km radii. These variables were used as predictors in the random forest model. Model was set to generate 5000 trees at one run and re-run ten times (creating in total 50,000 trees) to see the variation in the variable importance. We used cross-validation technique to evaluate the performance of the models. The R-squared value, which measured the proportion of variance in the observed parasitism explained by our model predictions. The decision tree was built on the predictors and target variable. We used “anova” method, which is used to solve regression task where the target variable is continuous or numeric. For the analysis, we used the ‘randomForest’^77^, ‘rpart'^78^ and ‘partykit'^79^ pacakages in Rstudio.

### Supplementary Information

Supplementary Tables.

## Data Availability

Data used in the study is available from the authors on a request.
