# Supplementary material for: Landscape complexity effects on Brassicogethes aeneus abundance and larval parasitism rate: a two-year field study
Source: Sci Rep. 2023 Dec 16;13:22373. doi: 10.1038/s41598-023-49690-1 (PMC10725428; doi:10.1038/s41598-023-49690-1)
Supplement: Supplementary file 1 — Supplementary Tables. [file 41598_2023_49690_MOESM1_ESM.pdf]

Landscape complexity effects on *Brassicogethes aeneus* abundance and larval parasitism rate: a two-year field study

Silva Vilumets, Riina Kaasik, Marjolein Lof, Gabriella Kovacs, John Holland, and Eve Veromann

Supplementary Table 1. The characteristics of the study region in 2014 hectares (ha): field size (ha), adjacent element type (herbaceous linear (HL)); woody linear (WL); another crop (CT), seminatural habitat (SNH) (%), arable land (%), arable land excluding permanent grassland (%), winter oilseed rape (OSR) (%), wheat (%), woody area (%), woody linear (%), herbaceous area (%), herbaceous linear (%), fallow area (%), permanent grassland (%).

ID	Year	Field size	Adjacent element type	SNH (%)	Arable land (%)	Arable land excluding permanent grassland (%)	OSR (%)	Wheat (%)	Woody area (%)	Woody linear (%)	Herbaceous area (%)	Herbaceous linear (%)	Fallow area (%)	Permanent grassland (%)
**LS1.HL**	2014	9.7	HL	22.9	59.1	28.8	6.8	1.3	13.3	0.7	7.6	1.4	0.0	30.3
**LS2.CT**	2014	37.8	CT	43.0	52.6	46.9	12.0	24.4	34.8	0.1	2.1	0.8	5.1	5.7
**LS3.CT**	2014	4.5	CT	68.7	26.4	25.8	9.5	5.0	59.4	0.0	8.1	0.6	0.5	0.6
**LS4.CT**	2014	9.5	CT	26.1	62.2	60.2	27.7	6.8	18.2	0.3	6.0	0.3	1.3	2.0
**LS5.HL**	2014	26.8	HL	33.7	36.2	33.5	8.9	14.4	20.1	0.3	12.0	1.3	0.0	2.7
**LS6.WL**	2014	12.4	WL	24.0	66.1	53.9	3.9	7.0	16.8	0.2	3.3	1.0	2.7	12.2
**LS7.HL**	2014	41.3	HL	28.0	69.2	66.9	23.6	20.1	25.6	0.0	1.5	0.9	0.0	2.3
**LS8.CT**	2014	52.3	CT	23.4	73.7	70.6	28.2	0.9	17.2	0.0	4.6	1.6	0.0	3.1
**LS9.WL**	2014	12	WL	30.7	51.7	24.0	3.8	0.0	13.8	1.7	13.3	1.9	0.0	27.7
**LS10.WL**	2014	11.5	WL	35.5	61.0	59.9	16.2	28.7	31.1	0.4	2.4	0.7	0.9	1.2
**LS11.HL**	2014	6.4	HL	34.2	59.3	56.6	10.5	24.1	27.6	0.8	3.1	0.8	1.9	2.7
**LS12.HL**	2014	6	HL	46.6	42.9	41.9	2.9	8.9	24.1	0.2	21.3	1.0	0.0	1.1
**LS13.HL**	2014	20.6	HL	37.0	57.3	56.3	6.6	33.3	29.4	0.1	6.7	0.8	0.0	1.0
**LS14.WL**	2014	73.8	WL	39.3	56.9	53.1	30.5	14.7	22.8	0.9	8.6	0.5	6.5	3.8
**LS15.CT**	2014	52.7	CT	47.7	46.8	43.8	26.1	8.8	31.2	0.3	8.4	0.5	7.2	3.0
**LS16.WL**	2014	43.7	WL	40.0	53.7	48.8	30.7	4.3	26.8	0.4	7.8	0.8	4.3	4.9
**LS17.WL**	2014	16.6	WL	75.9	20.2	18.5	5.3	0.7	61.3	0.9	11.7	0.7	1.4	1.7
**LS18.CT**	2014	10.5	CT	20.9	73.4	56.7	10.9	3.9	16.5	0.1	3.1	0.7	0.4	16.7
			**max**	75.9	73.7	70.6	30.7	33.3	61.3	1.7	21.3	1.9	7.2	30.3
			**min**	20.9	20.2	18.5	2.9	0.0	13.3	0.0	1.5	0.3	0.0	0.6
			**average**	37.7	53.8	47.0	14.7	11.5	27.2	0.4	7.3	0.9	1.8	6.8
			**HL average**	33.8	54.0	47.3	9.9	17.0	23.4	0.4	8.7	1.0	0.3	6.7
			**CT average**	38.3	55.9	50.7	19.1	8.3	29.6	0.1	5.4	0.7	2.4	5.2
			**WL average**	40.9	51.6	43.0	15.1	9.2	28.8	0.7	7.8	0.9	2.6	8.6

Supplementary Table 2. The characteristics of the study region in 2015 hectares (ha): field size(ha), adjacent element type (herbaceous linear (HL)); woody linear (WL); another crop (CT), seminatural habitat (SNH) (%), arable land (%), arable land excluding permanent grassland (%), winter oilseed rape OSR (%), wheat (%), woody area (%), woody linear (%), herbaceous area (%), herbaceous linear (%), fallow area (%), permanent grassland (%).

ID	Year	Field size	Adjacent element	SNH (%)	Arable land (%)	Arable land excluding permanent grassland (%)	OSR (%)	Wheat (%)	Woody area (%)	Woody linear (%)	Herbaceous area (%)	Herbaceous linear (%)	Fallow area (%)	Permanent grassland (%)
**LS1.HL**	2015	9.7	CT	11.3	82.7	82.7	14.0	16.7	7.6	0.5	2.0	1.3	0.0	0.1
**LS2.CT**	2015	37.8	CT	24.5	70.3	69.9	18.7	26.7	20.6	0.4	1.7	1.8	0.0	0.4
**LS3.CT**	2015	4.5	CT	43.0	53.1	53.1	19.9	5.7	30.0	0.7	3.1	0.3	9.0	0.0
**LS4.CT**	2015	9.5	CT	43.1	52.8	52.5	10.7	10.9	29.4	0.8	4.4	1.1	7.4	0.2
**LS5.HL**	2015	26.8	CT	39.5	54.5	54.5	11.7	0.2	33.4	0.2	4.7	1.1	0.0	0.0
**LS6.WL**	2015	12.4	CT	43.6	47.0	47.0	5.4	12.9	30.3	0.8	7.2	1.9	3.5	0.0
**LS7.HL**	2015	41.3	HL	22.2	68.1	58.8	12.8	38.9	16.1	0.4	3.4	2.3	0.0	9.3
**LS8.CT**	2015	52.3	HL	14.5	68.1	68.1	15.1	0.1	1.4	1.0	9.0	2.3	0.7	0.0
**LS9.WL**	2015	12	HL	29.2	67.2	67.2	19.1	8.8	25.1	0.0	0.6	3.4	0.0	0.0
**LS10.WL**	2015	11.5	HL	34.9	58.7	56.7	16.8	24.5	14.8	0.3	6.8	1.9	11.0	2.0
**LS11.HL**	2015	6.4	HL	47.7	48.8	48.8	11.9	14.9	40.7	0.1	3.4	2.0	1.4	0.0
**LS12.HL**	2015	6	HL	52.0	44.7	40.4	4.8	13.2	47.6	0.3	1.6	2.3	0.3	4.3
**LS13.HL**	2015	20.6	WL	24.1	70.0	70.0	29.6	23.3	18.1	0.4	3.4	2.2	0.0	0.0
**LS14.WL**	2015	73.8	WL	22.9	73.0	71.2	21.6	33.4	16.4	0.1	4.8	1.3	0.3	1.8
**LS15.CT**	2015	52.7	WL	36.8	53.7	37.7	9.6	1.6	21.4	0.5	4.4	1.2	9.2	16.0
**LS16.WL**	2015	43.7	WL	39.2	40.3	40.3	15.7	0.0	17.4	0.2	11.0	1.7	8.9	0.0
**LS17.WL**	2015	16.6	WL	44.1	45.2	43.1	15.7	15.1	29.2	0.7	10.2	1.4	2.6	2.1
**LS18.CT**	2015	10.5	WL	51.1	45.6	45.6	30.3	9.3	39.8	0.2	9.7	1.5	0.0	0.0
			**max**	52.0	82.7	82.7	30.3	38.9	47.6	1.0	11.0	3.4	11.0	16.0
			**min**	11.3	40.3	37.7	4.8	0.0	1.4	0.0	0.6	0.3	0.0	0.0
			**average**	34.7	58.0	56.0	15.7	14.2	24.4	0.4	5.1	1.7	3.0	2.0
			**HL average**	33.4	59.3	56.7	13.4	16.7	24.3	0.4	4.2	2.4	2.2	2.6
			**CT average**	34.2	60.1	60.0	13.4	12.2	25.2	0.5	3.8	1.2	3.3	0.1
			**WL average**	36.4	54.6	51.3	20.4	13.8	23.7	0.4	7.2	1.6	3.5	3.3

Supplementary Table 3. Mean (± SE) number of *Brassicogethes aeneus* per WOSR plant (collected using plant taping method) during bud stage of plants (BBCH 50–59), the parasitism rates (%) of *B. aeneus* larvae per field, and the total proportions (%) of semi-natural habitats (SNH) around the focal field within 1km radius, in 2014 and 2015.

Year	Adjacent habitat	Mean number of *B. aeneus* per plant (± SE)	Parasitism (%) (± SE)	Total SNH (%)
**2014**	Another crop	0.18 ± 0.08	53.98 ± 11.65	43.00
**2014**	Another crop	0.18 ± 0.05	91.67 ± 8.33	68.67
**2014**	Another crop	0.08 ± 0.03	93.75 ± 6.25	26.09
**2014**	Another crop	0.78 ± 0.12	83.89 ± 4.92	23.40
**2014**	Another crop	0.08 ± 0.03	85.00 ± 8.06	47.70
**2014**	Another crop	0.27 ± 0.07	51.60 ± 11,39	20.89
**2014**	Herbaceous linear SNH	0.03 ± 0.01	54.03 ± 15.66	22.91
**2014**	Herbaceous linear SNH	0.23 ± 0.07	55.00 ± 15.12	33.72
**2014**	Herbaceous linear SNH	0.35 ± 0.09	85.,32 ± 6.09	28.04
**2014**	Herbaceous linear SNH	0.24 ± 0.06	74.39 ± 6.05	34.18
**2014**	Herbaceous linear SNH	0.12 ± 0.03	74.75 ± 8.04	46.63
**2014**	Herbaceous linear SNH	0.18 ± 0.07	70.00 ± 10.18	37.04
**2014**	Woody linear SNH	0.32 ± 0.07	55.00 ± 15.12	24.02
**2014**	Woody linear SNH	0.80 ± 0.15	57.70 ± 11.06	30.68
**2014**	Woody linear SNH	0.11 ± 0.03	46.06 ± 13.62	35.51
**2014**	Woody linear SNH	0.07 ± 0.03	60.04 ± 9.00	39.31
**2014**	Woody linear SNH	0.12 ± 0,05	59.84 ± 10.94	40.04
**2014**	Woody linear SNH	0.49 ± 0.12	75.45 ± 13.39	75.91
**2015**	Another crop	0.19 ± 0.06	41.67 ± 14.43	11.30
**2015**	Another crop	0.54 ± 0.08	85.81 ± 7.10	24.46
**2015**	Another crop	1.86 ± 0.37	44.83 ± 9.19	43.00
**2015**	Another crop	0.19 ± 0.04	94.29 ± 5.71	43.14
**2015**	Another crop	0.17 ± 0.04	92.86 ± 7.14	39.46
**2015**	Another crop	0.39 ± 0.08	90.19 ± 4.96	43.64
**2015**	Herbaceous linear SNH	0.53 ± 0.08	100 ± 0	22.21
**2015**	Herbaceous linear SNH	0.59 ± 0.14	0 ± 0	14.46
**2015**	Herbaceous linear SNH	0.49 ± 0.07	0 ± 0	29.20
**2015**	Herbaceous linear SNH	0.24 ± 0.05	93.75 ± 6.25	34.92
**2015**	Herbaceous linear SNH	0.82 ± 0.15	45.09 ± 10.49	47.65
**2015**	Herbaceous linear SNH	0.87 ± 0.11	80.96 ± 8.36	52.04

**2015**	Woody linear SNH	0.32 ± 0.06	75.00 ± 25.00	24.07
**2015**	Woody linear SNH	0.34 ± 0.07	76.00 ± 19.39	22.88
**2015**	Woody linear SNH	0.4 ± 0.07	92.86 ± 7.14	36.81
**2015**	Woody linear SNH	0.32 ± 0.06	25.00 ± 25.00	39.23
**2015**	Woody linear SNH	0.58 ± 0.08	100 ± 0	44.13
**2015**	Woody linear SNH	0.11 ± 0.03	100 ± 0	51.12

Supplementary Table 4. Correlations between the total abundance of *Brassicogethes aeneus* in 2014 and landscape characteristics within the 1 km radius of the focal field. The first value in the table represents the correlation coefficient (r) and the second value represents the p-value.

	B. aeneus	Seminatural habitat	Arable land	Arable land without grassland	Oilseed rape	Wheat	Grassland	Woody area	Woody linear	Herbaceous area	Herbaceous linear	Fallow area
B. aeneus		-0.08<br>0.7558	0.08<br>0.7610	-0.06<br>0.8110	-0.15<br>0.5639	-0.36<br>0.1424	0.23<br>0.3632	-0.07<br>0.7874	0.31<br>0.2138	0.04<br>0.8711	0.68<br>0.0020	-0.37<br>0.1337
Seminatural habitat	-0.08<br>0.7558		-0.89<br>0.0000	-0.60<br>0.0090	-0.17<br>0.4936	-0.06<br>0.7992	-0.44<br>0.0659	0.93<br>0.0000	0.09<br>0.7173	0.39<br>0.1068	-0.37<br>0.1312	0.20<br>0.4311
Arable land	0.08<br>0.7610	-0.89<br>0.0000		0.82<br>0.0000	0.35<br>0.1493	0.17<br>0.4988	0.25<br>0.3196	-0.75<br>0.0004	-0.22<br>0.3816	-0.60<br>0.0080	0.13<br>0.6057	-0.05<br>0.8577
Arable land without grassland	-0.06<br>0.8110	-0.60<br>0.0090	0.82<br>0.0000		0.55<br>0.0179	0.43<br>0.0720	-0.35<br>0.1528	-0.43<br>0.0781	-0.50<br>0.0341	-0.60<br>0.0078	-0.23<br>0.3542	0.07<br>0.7796
Oilseed rape	-0.15<br>0.5639	-0.17<br>0.4936	0.35<br>0.1493	0.55<br>0.0179		-0.02<br>0.9497	-0.35<br>0.1510	-0.14<br>0.5751	-0.20<br>0.4258	-0.33<br>0.1880	-0.35<br>0.1576	0.50<br>0.0362
Wheat	-0.36<br>0.1424	-0.06<br>0.7992	0.17<br>0.4988	0.43<br>0.0720	-0.02<br>0.9497		-0.45<br>0.0582	0.07<br>0.7880	-0.23<br>0.3657	-0.37<br>0.1264	-0.33<br>0.1825	0.09<br>0.7366
Grassland	0.23<br>0.3632	-0.44<br>0.0659	0.25<br>0.3196	-0.35<br>0.1528	-0.35<br>0.1510	-0.45<br>0.0582		-0.50<br>0.0338	0.49<br>0.0400	0.04<br>0.8847	0.61<br>0.0077	-0.20<br>0.4375
Woody area	-0.07<br>0.7874	0.93<br>0.0000	-0.75<br>0.0004	-0.43<br>0.0781	-0.14<br>0.5751	0.07<br>0.7880	-0.50<br>0.0338		-0.08<br>0.7457	0.07<br>0.7678	-0.45<br>0.0578	0.11<br>0.6749
Woody linear	0.31<br>0.2138	0.09<br>0.7173	-0.22<br>0.3816	-0.50<br>0.0341	-0.20<br>0.4258	-0.23<br>0.3657	0.49<br>0.0400	-0.08<br>0.7457		0.35<br>0.1596	0.34<br>0.1698	0.08<br>0.7484
Herbaceous area	0.04<br>0.8711	0.39<br>0.1068	-0.60<br>0.0080	-0.60<br>0.0078	-0.33<br>0.1880	-0.37<br>0.1264	0.04<br>0.8847	0.07<br>0.7678	0.35<br>0.1596		0.24<br>0.3385	-0.14<br>0.5742
Herbaceous linear	0.68<br>0.0020	-0.37<br>0.1312	0.13<br>0.6057	-0.23<br>0.3542	-0.35<br>0.1576	-0.33<br>0.1825	0.61<br>0.0077	-0.45<br>0.0578	0.34<br>0.1698	0.24<br>0.3385		-0.47<br>0.0514
Fallow area	-0.37<br>0.1337	0.20<br>0.4311	-0.05<br>0.8577	0.07<br>0.7796	0.50<br>0.0362	0.09<br>0.7366	-0.20<br>0.4375	0.11<br>0.6749	0.08<br>0.7484	-0.14<br>0.5742	-0.47<br>0.0514	

Supplementary Table 5. Correlations between the total abundance of *Brassicogethes aeneus* in 2015 and landscape characteristics within the 1 km radius of the focal field. The first value in the table represents the correlation coefficient (r) and the second value represents the p-value.

	B. aeneus	Seminatural habitat	Arable land	Arable land without grassland	Oilseed rape	Wheat	Grassland	Woody area	Woody linear	Herbaceous area	Herbaceous linear	Fallow area
B. aeneus		0.3825<br>0.22	0.5024<br>-0.17	0.5418<br>-0.15	0.7337<br>-0.09	0.6673<br>-0.11	0.9569<br>-0.01	0.3260<br>0.25	0.4875<br>0.17	0.2901<br>-0.26	0.3639<br>-0.23	0.4665<br>0.18
Seminatural habitat	0.3825<br>0.22		0.0000<br>-0.92	0.0000<br>-0.86	0.3942<br>-0.21	0.1497<br>-0.35	0.9602<br>-0.01	0.0000<br>0.89	0.5951<br>-0.13	0.3511<br>0.23	0.3727<br>-0.22	0.2103<br>0.31
Arable land	0.5024<br>-0.17	0.0000<br>-0.92		0.0000<br>0.95	0.2517<br>0.29	0.0387<br>0.49	0.9118<br>-0.03	0.0014<br>-0.69	0.9767<br>-0.01	0.0335<br>-0.50	0.4639<br>0.18	0.0854<br>-0.42
Arable land without grassland	0.5418<br>-0.15	0.0000<br>-0.86	0.0000<br>0.95		0.1280<br>0.37	0.0782<br>0.43	0.1541<br>-0.35	0.0051<br>-0.63	0.9162<br>-0.03	0.0760<br>-0.43	0.4711<br>0.18	0.0499<br>-0.47
Oilseed rape	0.7337<br>-0.09	0.3942<br>-0.21	0.2517<br>0.29	0.1280<br>0.37		0.4292<br>0.20	0.1878<br>-0.33	0.4579<br>-0.19	0.2529<br>-0.28	0.6045<br>0.13	0.9951<br>0.00	0.4533<br>-0.19
Wheat	0.6673<br>-0.11	0.1497<br>-0.35	0.0387<br>0.49	0.0782<br>0.43	0.4292<br>0.20		0.6791<br>0.10	0.4581<br>-0.19	0.4496<br>-0.19	0.2020<br>-0.32	0.4966<br>0.17	0.2256<br>-0.30
Grassland	0.9569<br>-0.01	0.9602<br>-0.01	0.9118<br>-0.03	0.1541<br>-0.35	0.1878<br>-0.33	0.6791<br>0.10		0.8119<br>-0.06	0.8100<br>0.06	0.6051<br>-0.13	0.9159<br>-0.03	0.3358<br>0.24
Woody area	0.3260<br>0.25	0.0000<br>0.89	0.0014<br>-0.69	0.0051<br>-0.63	0.4579<br>-0.19	0.4581<br>-0.19	0.8119<br>-0.06		0.2247<br>-0.30	0.6378<br>-0.12	0.6955<br>-0.10	0.7637<br>-0.08
Woody linear	0.4875<br>0.17	0.5951<br>-0.13	0.9767<br>-0.01	0.9162<br>-0.03	0.2529<br>-0.28	0.4496<br>-0.19	0.8100<br>0.06	0.2247<br>-0.30		0.3013<br>0.26	0.3093<br>-0.25	0.3378<br>0.24
Herbaceous area	0.2901<br>-0.26	0.3511<br>0.23	0.0335<br>-0.50	0.0760<br>-0.43	0.6045<br>0.13	0.2020<br>-0.32	0.6051<br>-0.13	0.6378<br>-0.12	0.3013<br>0.26		0.5353<br>-0.16	0.2552<br>0.28
Herbaceous linear	0.3639<br>-0.23	0.3727<br>-0.22	0.4639<br>0.18	0.4711<br>0.18	0.9951<br>0.00	0.4966<br>0.17	0.9159<br>-0.03	0.6955<br>-0.10	0.3093<br>-0.25	0.5353<br>-0.16		0.0840<br>-0.42
Fallow area	0.4665<br>0.18	0.2103<br>0.31	0.0854<br>-0.42	0.0499<br>-0.47	0.4533<br>-0.19	0.2256<br>-0.30	0.3358<br>0.24	0.7637<br>-0.08	0.3378<br>0.24	0.2552<br>0.28	0.0840<br>-0.42	
